# Nutritional Care of the Frail Patient with Heart Failure: Evidence, Uncertainties and Research Priorities

**DOI:** 10.3390/nu18213527

**Published:** 2026-10-26

**Authors:** Adrian Kwaśny, Małgorzata Słoma-Krześlak, Izabella Uchmanowicz, Christopher S. Lee, Ercole Vellone, Raúl Juárez-Vela, Stanisław Surma, Mateusz Labudda, Michał Czapla

**Affiliations:** 1 Institute of Dietetics, The Academy of Business and Health Science, 90-361 Lodz, Poland; 2 Department Human Nutrition, Department of Dietetics, Faculty of Public Health in Bytom, Medical University of Silesia in Katowice, ul. Jordana 19, 41-808 Zabrze, Poland; msloma-krzeslak@sum.edu.pl; 3 Department of Nursing, Faculty of Nursing and Midwifery, Wroclaw Medical University, 51-618 Wroclaw, Poland; izabella.uchmanowicz@umw.edu.pl (I.U.); ercole.vellone@uniroma2.it (E.V.); 4 Centre for Cardiovascular Health, Edinburgh Napier University, Sighthill Campus, Edinburgh EH14 1DJ, UK; 5 Boston College William F. Connell School of Nursing, Chestnut Hill, MA 02467, USA; christopher.lee.8@bc.edu; 6 Department of Biomedicine and Prevention, Tor Vergata University of Rome, 00133 Rome, Italy; 7 Group of Research in Care (GRUPAC), Faculty of Health Science, University of La Rioja, 26006 Logroño, Spain; raul.juarez@unirioja.es (R.J.-V.); michal.czapla@umw.edu.pl (M.C.); 8 Institute of Neurosciences of Castile and León (INCYL), University of Salamanca, Biomedical Research Institute of Salamanca (IBSAL), 37007 Salamanca, Spain; 9 Department of Internal Medicine and Clinical Pharmacology, Medical University of Silesia, 40-055 Katowice, Poland; stanislaw.surma@sum.edu.pl; 10 Department of Biochemistry and Microbiology, Faculty of Biology and Biotechnology, Warsaw University of Life Sciences-SGGW, Nowoursynowska 159, 02-776 Warsaw, Poland; mateusz_labudda@sggw.edu.pl; 11 Division of Scientific Research and Innovation in Emergency Medical Service, Department of Emergency Medical Service, Faculty of Nursing and Midwifery, Wroclaw Medical University, 51-618 Wroclaw, Poland; 12 Nursing Care and Education Research Group (GRIECE), Department of Nursing, University of Valencia, 46010 Valencia, Spain

**Keywords:** heart failure, frailty, malnutrition, sarcopenia, cachexia, nutritional assessment, nutritional support, protein intake

## Abstract

Frailty is highly prevalent in patients with heart failure (HF) and frequently coexists with malnutrition, sarcopenia, cachexia, and obesity, creating substantial challenges for nutritional assessment and management. These conditions overlap clinically but represent distinct biological phenotypes and should not be treated as interchangeable constructs. This narrative review critically synthesizes current evidence on nutritional care in patients with HF and frailty, focusing on nutritional assessment, energy and protein requirements, areas of alignment and conflict between HF- and frailty-oriented recommendations, and the effects of nutritional interventions on frailty-related outcomes. Nutritional assessment should be multidimensional, as congestion, inflammation, altered body composition, and renal dysfunction may limit the interpretability of body weight, BMI, biochemical indices, and screening tools. Current recommendations for energy and protein intake remain largely extrapolated from geriatric, multimorbidity, renal, or general HF populations, and no universally validated targets exist for frail patients with HF. Dietary management therefore requires balancing potentially competing priorities, including congestion control, preservation of muscle mass and function, adequate nutrient intake, renal limitations, and, in obesity-related HF, intentional fat loss without excessive loss of lean tissue. Available interventional evidence suggests that individualized nutritional support may improve nutritional status and selected functional outcomes, but effects on frailty as an integrated syndrome remain uncertain. Overall, nutritional care in HF with frailty should move towards phenotype-directed, multidisciplinary strategies integrating nutrition, physical activity, rehabilitation, and longitudinal assessment. Future trials should standardize frailty definitions, clinically relevant outcomes, and nutrition-related targets.

## 1. Introduction

Heart failure (HF) is increasingly recognized as a systemic syndrome in which cardiovascular dysfunction coexists with abnormalities in nutritional status, skeletal muscle, physical function, and physiological reserve. A recent meta-analysis of 23 studies involving 6287 patients with chronic HF estimated the prevalence of frailty at 39% (95% CI 33–45%), although substantial between-study heterogeneity was observed (I^2^ = 96.2%) [1]. Clinical setting also influences the observed burden of frailty; in an earlier meta-analysis, frailty was present in 59.0% of patients hospitalized for HF exacerbation and 32.3% of outpatients [1,2]. Frailty is particularly common in HF and is associated with impaired quality of life, disability, recurrent hospitalization, and increased mortality [3,4]. At the same time, malnutrition, sarcopenia, and cachexia frequently coexist with frailty, although these conditions represent distinct clinical and biological entities rather than interchangeable manifestations of the same process [3,5]. Distinguishing these phenotypes is clinically important because each involves different nutritional priorities, making a uniform nutritional strategy potentially inappropriate for patients with HF and frailty.

Nutritional abnormalities are clinically relevant across the HF spectrum. A recent systematic review and meta-analysis reported a high prevalence of malnutrition in patients with HF, while also demonstrating substantial heterogeneity related to the diagnostic approach used [6]. This is particularly important because congestion, inflammation, renal dysfunction, and changes in body composition can substantially complicate nutritional assessment and interpretation of conventional anthropometric or biochemical measures [3,5]. Nutritional management in HF with frailty involves several competing therapeutic priorities, including sodium and fluid restriction versus adequate nutritional intake, increased protein requirements versus renal dysfunction, and intentional weight loss in obesity versus preservation of skeletal muscle mass and functional reserve. These competing priorities highlight the challenges of developing nutritional strategies that simultaneously address HF management and frailty-related vulnerabilities.

Despite increasing recognition of these problems, evidence guiding nutritional management in patients with both HF and frailty remains limited. Current recommendations are largely derived from studies of HF, malnutrition, sarcopenia, geriatrics, or multimorbidity separately, and the optimal integration of nutritional support with exercise, rehabilitation, and contemporary HF therapy remains uncertain [5,7]. The added value of this review lies in integrating HF-specific and frailty-oriented nutritional recommendations, critically examining their areas of agreement and conflict, and distinguishing evidence directly derived from patients with HF and frailty from findings extrapolated from related populations.

Accordingly, this narrative review aims to critically synthesize current evidence on nutritional care in patients with HF and frailty, focusing on nutritional assessment, energy and protein requirements, and the effects of nutritional interventions on frailty-related outcomes. Particular attention is given to distinguishing evidence derived directly from patients with both HF and frailty from findings extrapolated from broader HF, geriatric, renal, sarcopenia, or malnutrition populations. Beyond summarizing the available evidence, we aim to identify areas of agreement between existing recommendations, unresolved therapeutic conflicts, and priorities for future research.

## 2. Literature Search

This narrative review was informed by a literature search conducted between July and September 2026 in MEDLINE (via PubMed), EMBASE, and the Cochrane Central Register of Controlled Trials (CENTRAL). Relevant publications identified during manuscript preparation and subsequent revisions were also considered to incorporate emerging evidence. Search terms covered heart failure, frailty, malnutrition, sarcopenia, cachexia, obesity, nutritional assessment, body composition, energy and protein requirements, dietary recommendations, nutritional interventions, exercise, rehabilitation, and clinical outcomes. Relevant English-language original studies, systematic reviews, meta-analyses, clinical practice guidelines, and consensus statements were considered. Where evidence specifically addressing patients with both heart failure and frailty was limited, findings from related populations were included when clinically relevant. Literature selection was narrative and guided by relevance to the review objectives, with priority given to recent guidelines, consensus statements, evidence syntheses, and clinically relevant interventional studies. Earlier landmark publications were retained where appropriate. Evidence was critically appraised narratively, considering study design, sample size, methodological limitations, consistency and precision of findings, clinical relevance, and the directness of evidence to patients with both HF and frailty. No formal risk-of-bias assessment or GRADE evaluation was performed. No formal systematic screening or study selection procedures were performed. Accordingly, this review should be interpreted as a critical narrative synthesis rather than a systematic or exhaustive review of the literature. The scope of this review encompasses patients with HF and frailty, while acknowledging the heterogeneity of frailty definitions and assessment methods across studies. No single frailty instrument or diagnostic threshold was applied as an eligibility criterion. Where direct evidence from patients with formally assessed frailty was unavailable, relevant findings from older adults with HF or populations characterized by malnutrition, sarcopenia, or cachexia were considered. Such evidence is interpreted as indirect and should not be assumed to apply equally to patients with both HF and frailty.

## 3. Malnutrition, Sarcopenia, Cachexia and Frailty: Conceptual Distinctions

Malnutrition, sarcopenia, cachexia, and frailty syndrome are common, partially overlapping, yet biologically and clinically distinct phenotypes in patients with heart failure (HF) [3,8,9]. Their coexistence reflects the interplay of chronic disease, inflammation, anabolic–catabolic imbalance, reduced physical activity, inadequate nutrient intake, and the progressive loss of physiological reserves [3,8,9]. However, they should not be viewed merely as successive stages along a single continuum of wasting. It is more useful to regard them as intersecting axes of biological vulnerability: substrate deficiency, muscle dysfunction, pathological catabolism, and the loss of multisystem reserve [3,8,9].

Malnutrition is primarily characterized by the insufficient availability or utilization of nutritional substrates, leading to adverse changes in body composition and physiological function [10]. The 2025 updated consensus from the Global Leadership Initiative on Malnutrition (GLIM) maintained a two-step diagnostic framework: following the identification of nutritional risk, a diagnosis requires at least one phenotypic criterion—unintentional weight loss, low BMI, or reduced muscle mass—and at least one etiologic criterion, involving reduced food intake/assimilation or inflammation/disease burden [10]. In HF, the application of anthropometric criteria is particularly challenging, as fluid retention can mask the loss of lean body mass, while a normal or high BMI does not rule out significant muscle depletion [3,8]. This is not a minor issue: a meta-analysis of 91 studies identified malnutrition in 44.6% of HF patients; however, extreme heterogeneity (I^2^ = 99.1%), the dependence of results on the diagnostic tool used, and very low certainty of evidence demonstrate the extent to which prevalence rates depend on the definition of the phenotype under study [6].

Sarcopenia shifts the focus from nutritional substrates to the integrity of skeletal muscle. The 2024 consensus from the Global Leadership Initiative in Sarcopenia (GLIS) defines sarcopenia as a generalized skeletal muscle disorder, with conceptual components comprising reduced muscle mass, muscle strength, and muscle-specific strength [11]. Notably, impaired physical performance is considered primarily a consequence of sarcopenia rather than a constitutive element thereof. The definition should also not vary based on age, care setting, or the underlying disease, including HF [11]. Thus, HF does not constitute a distinct “cardiac sarcopenia” but rather creates a biological environment conducive to the accelerated loss of muscle mass and function through reduced activity, inflammation, and a predominance of catabolic processes [3,8,9].

This distinction is particularly important in patients with normal or increased body weight. Sarcopenia does not require a loss of total body weight and can coexist with obesity [3,8]. Thus, a high BMI is not the opposite of muscle wasting, and stable body weight can mask significant changes in body composition [3,8]. Body weight, nutritional status, and muscle integrity therefore describe three related but distinct biological characteristics.

Cardiac cachexia represents yet another process. It is an active, disease-associated catabolic syndrome characterized by muscle loss and unintentional, edema-free weight loss, which can occur with either insufficient or seemingly adequate macronutrient intake [9]. A contemporary proposed definition of cardiac cachexia adopts a threshold of ≥5% unintentional edema-free weight loss relative to pre-HF weight, accompanied by muscle loss; however, the lack of a uniform definition remains a significant limitation in research on this phenotype [9]. Cachexia is therefore neither “severe sarcopenia” nor a simple consequence of malnutrition. In the SICA-HF study, only 6.7% of men met the criteria for both cachexia and sarcopenia, and this overlapping phenotype was associated with the greatest impairment in strength, function, and quality of life [9].

Recent data are further shifting the understanding of cachexia from passive weight loss toward active metabolic dysregulation. In 344 patients with advanced HFrEF, higher GDF-15 levels were associated with anorexia, cachexia, increased congestion and right ventricular dysfunction, and a metabolic profile indicative of heightened catabolism [12]. While this does not prove a causal role for GDF-15, it suggests a potential link between hemodynamics, neurohumoral signaling, appetite, and energy metabolism [12]. The clinical significance of cachexia is confirmed by a 2024 meta-analysis, which linked the condition to an approximately 60% higher risk of all-cause mortality (HR 1.60; 95% CI 1.31–1.95), with the high heterogeneity in estimated prevalence further highlighting the issue of definition [13].

Frailty encompasses an even broader level of biological vulnerability. A 2025 international consensus on HF defines it as a multidimensional, dynamic, and potentially reversible state—associated with HF but independent of age—that increases vulnerability to stressors and the risk of adverse outcomes [14]. The Heart Failure Frailty Score, developed by 54 experts, incorporates 22 variables across four domains—clinical, functional, psycho-cognitive, and social—demonstrating why frailty cannot be reduced to sarcopenia or malnutrition [14]. At the same time, the overlap between HF symptoms and frailty components increases the risk of misclassification, particularly when using non-specific tools based primarily on physical performance [14,15]. Across these phenotypes, congestion may obscure weight and muscle loss, obesity may mask nutritional and muscular deficits, and HF-related exercise intolerance may complicate the interpretation of physical performance, highlighting the need for multidimensional assessment.

**Figure 1:**
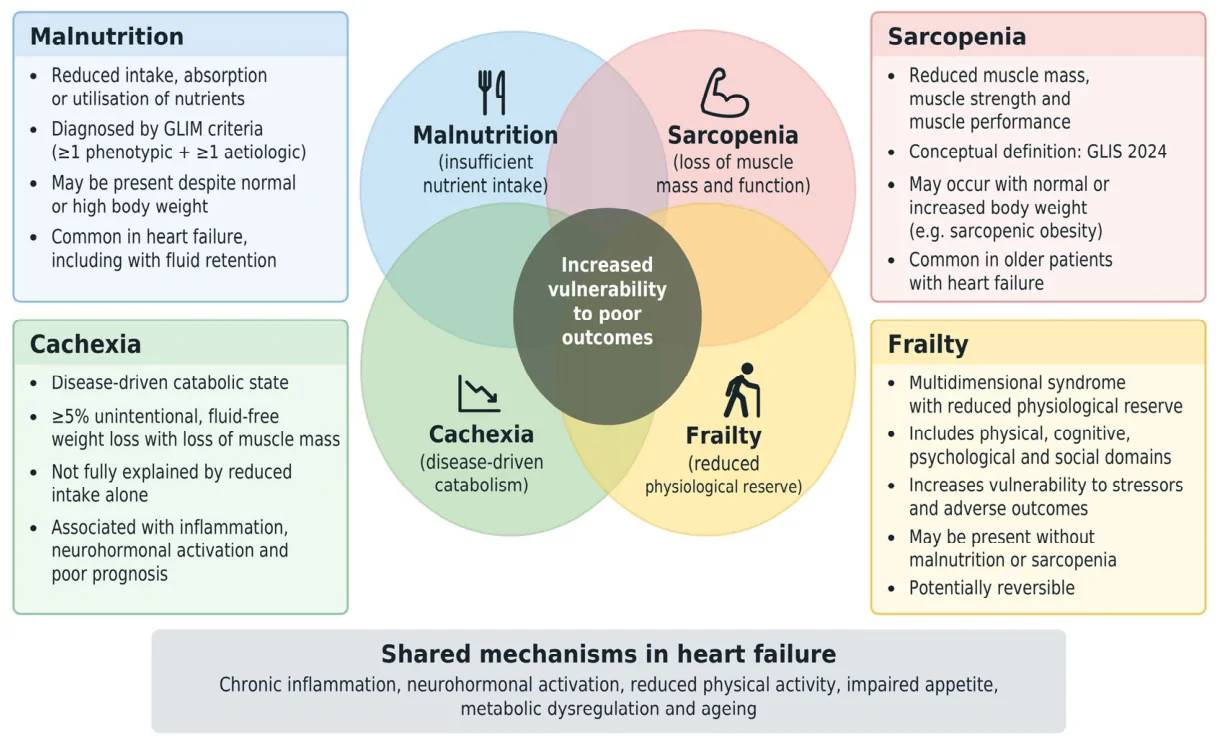
Malnutrition, sarcopenia, cachexia and frailty in heart failure: distinct but overlapping phenotypes.

Distinguishing between these four phenotypes is, therefore, not merely a matter of semantics. Malnutrition primarily identifies a deficiency or improper utilization of substrates; sarcopenia—muscle insufficiency; cachexia—disease-driven catabolism; and frailty—a loss of multisystem reserve and resilience to stressors [3,9,10,11,14] (Figure 1). A single patient may occupy different positions along each of these axes, and their relative importance may shift over the course of HF [3,8]. Rather than seeking a single overarching label, it is clinically more useful to phenotype co-existing deficits and identify the dominant, potentially reversible mechanism. In clinical practice, this approach involves initial screening for nutritional risk and frailty, followed by assessment of muscle mass and strength, weight trajectory, and clinical features of disease-related catabolism. These complementary assessments may help identify overlapping phenotypes and guide further nutritional evaluation, as discussed in Section 4. This perspective translates into distinct therapeutic priorities: nutritional repletion in malnutrition, preservation or restoration of muscle mass and function through adequate nutrition and resistance exercise in sarcopenia, prevention of further catabolic loss in cachexia, and multidomain nutritional and functional support in frailty [3,8,9,14]. These distinctions provide a basis for the individualized nutritional assessment and interventions discussed in the following sections.

## 4. Nutritional Assessment in Frail Patients with HF

Nutritional abnormalities are common in patients with HF and are closely intertwined with frailty and adverse clinical outcomes [16,17]. However, nutritional assessment in this population remains challenging, as HF itself can substantially affect several variables used to determine nutritional status. Congestion may distort body weight and BMI, while systemic inflammation and disease severity can influence biochemical markers and body composition, potentially causing markers of HF severity to be misinterpreted as indicators of malnutrition [18]. Moreover, excess body weight does not exclude nutritional impairment or reduced muscle mass, particularly in older and frail patients with HF [18,19]. Consequently, nutritional assessment in frail patients with HF requires a multidimensional approach that distinguishes nutritional risk, established malnutrition, and prognostic risk rather than relying on a single anthropometric or biochemical measure [5,17].

### 4.1. From Nutritional Screening to Comprehensive Assessment and Diagnosis

NRS-2002 and MUST provide rapid identification of patients at nutritional risk but rely substantially on weight and BMI, which may be distorted by congestion in HF. The limitations of weight-based assessment are particularly relevant in HF: BMI does not adequately capture body composition, including lean and skeletal muscle mass, and may be further confounded by oedema; importantly, a high BMI does not exclude malnutrition [20]. NRS-2002 has nonetheless demonstrated independent prognostic value in hospitalized patients with chronic HF [21], whereas HF-specific evidence for MUST remains comparatively limited. MNA-SF has been more extensively investigated in HF; in a systematic review including 25,141 patients, it emerged as a particularly promising screening instrument [22]. However, nutritional screening tools should not be interpreted as diagnostic measures. In a direct comparison of MNA-SF, GNRI and CONUT in 150 HF outpatients, agreement was only slight between MNA-SF and GNRI (κ = 0.16) and between GNRI and CONUT (κ = 0.11), and absent between MNA-SF and CONUT (κ = −0.09) [23]. These findings indicate that different instruments may capture distinct dimensions of nutritional risk rather than measuring an identical underlying construct.

Patients identified as being at nutritional risk require more comprehensive assessment. MNA and SGA incorporate broader clinical, dietary, anthropometric and functional information, whereas GLIM provides a consensus diagnostic framework requiring at least one phenotypic criterion (weight loss, low BMI or reduced muscle mass) and one etiologic criterion (reduced food intake or assimilation, or inflammation/disease burden) [24]. In ambulatory patients with chronic HF, MNA, but not SGA, independently predicted mortality [25]. Evidence regarding GLIM is less consistent and appears setting-dependent: among 151 chronic HF outpatients, MNA but not GLIM independently predicted five-year mortality, cardiovascular mortality and recurrent HF hospitalization [26], whereas in 446 patients hospitalized with acute HF, GLIM-defined malnutrition independently predicted one-year all-cause death or HF readmission (HR 1.59, 95% CI 1.06–2.40), while nutritional risk identified by MNA-SF did not [27]. Thus, screening, comprehensive assessment and diagnostic approaches provide complementary rather than interchangeable information, and their relative performance may vary according to clinical setting and outcome considered.

### 4.2. Prognostic Nutritional Indices and the Modifying Role of Frailty

GNRI, CONUT and PNI have been widely investigated as prognostic nutritional indices in HF, with poorer scores consistently associated with mortality and other adverse clinical outcomes [22,28]. However, their prognostic value should not be equated with diagnostic validity for malnutrition. GNRI incorporates serum albumin and body weight, both of which may be influenced by congestion, inflammation and disease severity in HF. CONUT additionally incorporates total cholesterol and lymphocyte count, making its interpretation potentially sensitive to lipid-lowering therapy, immune status and systemic inflammation, while PNI is likewise based on albumin and lymphocyte count. Importantly, these indices have been associated not only with mortality but also with inflammatory markers, natriuretic peptides, myocardial injury markers and indices of volume overload in HF [28]. They may therefore reflect the combined effects of nutritional impairment, systemic inflammation and HF severity rather than nutritional status alone.

The presence of frailty may further modify their prognostic significance. In a prospective multicentre cohort of 1617 patients with HF aged ≥65 years, GNRI, CONUT and MNA-SF were all associated with the two-year composite outcome among frail patients in unadjusted analyses. After adjustment, CONUT retained a significant overall trend across categories, whereas GNRI showed an association in one category without a significant overall trend; MNA-SF was not independently associated with the outcome [17]. These findings suggest that the prognostic information provided by nutritional measures may differ according to frailty status and should therefore be interpreted within the broader clinical and functional phenotype rather than in isolation.

### 4.3. Beyond Nutritional Scores: Anthropometry, Body Composition and Integrated Assessment

Nutritional assessment in frail patients with HF should extend beyond screening scores and laboratory-based indices to include anthropometric, body-composition and functional measures [29,30]. Reliance on BMI alone is particularly problematic because excess body weight may coexist with nutritional impairment, reduced muscle mass and frailty. In a cohort of 200 older patients hospitalized with HF, frailty according to the Fried phenotype was present in 65%, while 36.5% were malnourished or at nutritional risk according to MNA; importantly, frailty was not significantly associated with BMI, calf circumference or arm circumference [29]. These findings illustrate that conventional anthropometric measures and frailty capture distinct, although clinically overlapping, dimensions of vulnerability in HF. The limitations of anthropometric classification are further illustrated by a cohort of 250 older adults hospitalized with acute decompensated HF, in whom the prevalence of obesity varied substantially according to the definition applied: 36.8% using BMI, 64.8% using waist circumference and 50.0% using a clinical obesity framework [31]. None of these obesity definitions was independently associated with hospital length of stay, underscoring that anthropometric classifications are not interchangeable and may provide limited information on clinical vulnerability when considered in isolation [31].

Interpretation of anthropometric and body-composition measures is further complicated by fluid status. During HF decompensation, congestion may alter body weight and derived anthropometric indices, while hydration status can also affect bioelectrical impedance measurements [32]. The timing of anthropometric assessment is therefore important; measurements obtained after clinical stabilization may reduce misclassification related to acute volume overload [31]. Consequently, body weight and BMI should be interpreted in relation to volume status and, where feasible, complemented by measures that characterize muscle quantity, strength and physical performance. Approaches such as dual-energy X-ray absorptiometry or bioelectrical impedance analysis may provide additional information on body composition, whereas handgrip strength and performance-based measures can help characterize the functional consequences of muscle impairment and frailty [33,34]. Contemporary frailty assessment in HF increasingly emphasizes such multidimensional evaluation rather than reliance on a single anthropometric or functional marker [33].

Taken together, no single nutritional measure adequately captures the nutritional phenotype of a frail patient with HF. In clinical practice, nutritional evaluation should integrate congestion and weight trajectory, nutritional risk screening, comprehensive assessment when indicated, and evaluation of muscle mass, strength, and physical performance, with biochemical and prognostic indices interpreted in the context of HF severity, inflammation, and renal function.

Screening tools, comprehensive assessment methods, diagnostic frameworks and prognostic nutritional indices serve different but complementary purposes, while anthropometric and body-composition measures require interpretation in the context of congestion, muscle status and physical frailty. The main characteristics, potential applications and limitations of commonly used nutritional assessment approaches in frail patients with HF are summarized in Table 1. Importantly, nutritional status, sarcopenia, and frailty should be assessed as distinct but interrelated clinical domains rather than inferred from BMI, nutritional screening scores, or isolated measures of physical performance.

## 5. Energy and Protein Requirements

No single energy requirement applies across heart failure (HF), and none has been prospectively validated for HF with frailty. The numerical recommendations discussed below originate from different clinical populations and methodological frameworks and should therefore not be interpreted as interchangeable or directly applicable to patients with both HF and frailty. Age, activity, inflammation, comorbidities and disease severity modify expenditure. Resting energy expenditure (REE), representing energy expended at rest, may be altered in HF, whereas total energy expenditure (TEE), which also accounts for physical activity and other components of daily energy expenditure, can remain low because of markedly reduced physical activity; resting metabolic abnormalities should therefore not be equated with uniformly increased total energy requirements [35,36]. Malnutrition, sarcopenia and cachexia impose different recovery needs; nutritional repletion alone should not be expected to reverse the multifactorial catabolic processes underlying cardiac cachexia [37,38].

Indirect calorimetry, when available, is preferable for assessing REE; estimating TEE additionally requires consideration of activity [39]. In 72 hospitalised patients with advanced HF, Sakamoto et al. measured REE but calculated TEE using an activity factor of 1.3. Mean REE was 25 kcal/kg/day overall and 28 kcal/kg/day in underweight participants. Their BMI-dependent equations require external validation because participants were relatively young and highly selected; measurements were obtained during haemodynamic stability, sometimes with inotropic support [40]. These findings caution against assuming that predictive equations accurately represent requirements in older patients with frailty.

When measurement is unavailable, HF-specific quantitative guidance remains limited. The 2025 Japanese Circulation Society/Japanese Heart Failure Society (JCS/JHFS) guideline cites observational REE values of 22–24 kcal/kg/day in selected Stage B/C populations and 18 kcal/kg/day in Stage D patients receiving left ventricular assist device support, with TEE estimated using an activity factor [41]. These are not validated targets for frail HF. ESPEN suggests approximately 27 kcal/kg/day for TEE in older polymorbid inpatients [39], whereas geriatric guidance uses 30 kcal/kg/day as an adjustable guiding value [42]. In severe underweight, the ESPEN polymorbidity guideline’s 30 kcal/kg estimate refers to REE, not a TEE ceiling [39]. Such values can inform initial estimates but do not establish HF-with-frailty targets; subsequent adjustment should reflect activity, tolerance and recovery goals [35,42].

The reference weight must also be explicit. Because oedema and effusions can obscure tissue loss, body weight should be interpreted in relation to congestion status and reassessed after decongestion; admission weight in patients with significant fluid overload should not automatically be used to calculate energy or protein requirements [37,38]. Actual weight multiplied by a fixed factor may overestimate requirements in obesity, but no universally validated HF-specific ideal or adjusted-weight correction exists. In practice, after substantial wasting, current dry weight, previous weight and the intended recovery trajectory should inform interpretation rather than prescribing automatically from historical or depleted weight [37,42].

Stable chronic HF permits adjustment towards maintenance or nutritional recovery. Decompensation requires reassessment of intake, perfusion and tolerance, but does not invariably entail critical illness. For critically ill patients, ESPEN recommends progressive feeding rather than immediate full replacement of estimated expenditure [43]. Extending this approach to every HF admission would exceed its evidence base. Both persistent underfeeding and overfeeding are undesirable; nominal targets should not displace assessment of achievable intake and clinical response.

Protein recommendations similarly differ in origin. The Heart Failure Society of America considers at least 1.1 g/kg/day reasonable for HF with malnutrition or cachexia, based on limited evidence [37]. JCS/JHFS 2025 similarly recommends at least 1.1 g/kg/day in HF without moderate-to-severe renal dysfunction and 1.2–1.5 g/kg/day in older patients [41]. The 2026 American Heart Association statement suggests 1.0–1.2 g/kg/day may be appropriate in cardiac cachexia [44]. ESPEN geriatric guidance advises at least 1.0 g/kg/day, while expert and polymorbidity guidance commonly use 1.2–1.5 g/kg/day during illness or malnutrition [39,42,45]. These higher ranges remain largely extrapolative for HF with frailty and should be conditional on renal function, energy adequacy and tolerance.

Kidney disease requires separate consideration. Kidney Disease: Improving Global Outcomes guidance suggests approximately 0.8 g/kg/day in stable chronic kidney disease (CKD) stages G3–G5 without dialysis, while allowing higher targets in older adults with frailty or sarcopenia [46]. During acute or critical illness without kidney replacement therapy (KRT), ESPEN suggests starting at 1.0 and increasing towards 1.3 g/kg/day if tolerated. Suggested ranges in critical illness are 1.3–1.5 g/kg/day with intermittent KRT and 1.5–1.7 g/kg/day with continuous or prolonged KRT; stable chronic haemodialysis inpatients require at least 1.2 g/kg/day [47]. These are renal recommendations, not validated HF targets. Protein should not be restricted merely to postpone KRT. The American Heart Association likewise identifies protein requirements during concurrent CKD and acute cardiovascular illness as unresolved, supporting individual assessment rather than automatic restriction or escalation [44].

Lower energy intake or estimated protein intake has been associated with worse outcomes, but anorexia, inflammation, congestion and disease severity can contribute to both inadequate intake and poor prognosis [48,49]. In 694 hospitalised patients, Dotare et al. found an adjusted association between lower estimated protein intake and mortality without CKD, but not with CKD, with a significant interaction [50]. This observational result cannot establish a renal-specific prescription. Individualised support reduced short-term mortality in a randomised trial’s HF subgroup, but tested a multicomponent strategy rather than an isolated energy or protein dose [51].

Adequate essential amino acid provision and combining nutrition with feasible resistance exercise are supported principally by geriatric evidence. Optimal protein quality, meal doses and distribution remain uncertain in HF with frailty [45]. Anabolic responses in experimental settings do not establish superior clinical outcomes from a particular meal schedule.

Severely malnourished patients require assessment of refeeding risk, cautious advancement, thiamine provision and monitoring of phosphate, potassium, magnesium and fluid balance [52]. The energy and protein values discussed above should therefore be regarded as initial estimates rather than fixed prescriptions. Nutritional targets require ongoing reassessment according to actual intake, congestion status, renal function, gastrointestinal tolerance, weight trajectory, muscle function, refeeding risk, and evolving clinical goals [37,42,47].

## 6. Alignment and Conflicts Between HF and Frailty Nutritional Recommendations

Nutritional management for a patient with HF and frailty syndrome requires reconciling partially competing therapeutic goals. The traditional approach to HF focused on controlling congestion through sodium restriction and—in selected patients—fluid restriction; conversely, management targeting frailty, malnutrition, and sarcopenia aims to maintain adequate energy and protein intake, preserve muscle mass, and sustain physical function [3,53,54,55]. Thus, the issue is not merely determining which nutrients to restrict or supplement. The central challenge lies in maintaining euvolemia without compromising nutritional intake, preserving muscle mass while respecting cardiorenal limitations, and—in cases of obesity—reducing excess adipose tissue without exacerbating the loss of lean body mass and functional reserve [3,53,54,55]. Such tensions highlight a fundamental limitation of current guidelines: most remain focused on a single disease, whereas a frail patient with HF is, by definition, biologically complex and often suffers from multiple comorbidities [53,55]. Importantly, recommendations regarding sodium and fluid restriction and protein intake are largely derived from broader HF, geriatric, renal, or multimorbidity populations rather than studies specifically involving patients with both HF and formally assessed frailty. Their application to this population therefore relies substantially on extrapolation and individualized clinical judgment.

Sodium serves as the most striking example of the evolution of this paradigm. While sodium restriction is physiologically justified in conditions characterized by sodium and water retention, contemporary studies do not confirm clinical benefits from routine, intensive restriction across the overall HF population [53,54]. In the SODIUM-HF trial, a reduction in sodium intake of approximately 27% did not lower the risk of the composite endpoint—comprising death, hospitalization, or emergency department visits due to cardiovascular causes—after 12 months, although moderate improvements in NYHA class and select quality-of-life indicators were observed [56]. The current consensus from the Heart Failure Association of the ESC emphasizes that there is no basis for intensive sodium restriction as a universal strategy in either chronic or acute HF [54].

This is particularly relevant in the context of frailty. Sodium restriction should not compromise the overall nutritional quality of the diet; modern guidelines emphasize the importance of a dietary pattern based on whole, minimally processed foods rather than focusing solely on a specific sodium intake threshold [53]. Consequently, for patients experiencing anorexia, malnutrition, or progressive weight loss, any further intensification of dietary restrictions must be evaluated within the broader context of meeting energy and nutritional requirements [3,53,55]. A 2025 JACC: Heart Failure review does not recommend sodium restriction for all HF patients, proposing guidance aimed at an intake of <2000 mg/day primarily for patients with severe congestion or uncontrolled hypertension [53]. For a patient with heart failure and frailty, the goal should therefore not be to achieve the lowest possible sodium intake, but rather to determine the minimum level of restriction necessary to control congestion without compromising the nutritional adequacy of the diet [53,54].

Fluid restriction should be individualised rather than routinely prescribed in stable HF [53,54]. In FRESH-UP, 504 patients with chronic HF were randomised to liberal fluid intake or a 1.5 L/day restriction; the primary health-status outcome did not differ significantly at 3 months, while restriction increased thirst [57]. These findings should not be generalised to severe congestion or hyponatremia. In frail patients, fluid advice should also consider meal intake, dehydration risk, renal function and diuretic treatment [3,53,54].

Regarding protein, the conflict runs in the opposite direction. Frailty and sarcopenia argue for maintaining adequate protein intake, whereas advanced chronic kidney disease (CKD) may necessitate restriction [3,46,47]. The Nutritional Management Guidelines for Sarcopenia and Frailty 2025 recommend adequate energy and protein intake for patients with chronic HF, combined with physical activity and treatment of the underlying condition; in the absence of moderate-to-severe renal impairment, they suggest 1.2–1.5 g of protein/kg/day and 25–30 kcal/kg of standard body weight/day [55]. At the same time, the authors explicitly highlight the low certainty of the evidence and the lack of an established nutritional strategy tailored to the stage or severity of HF [55].

ESPEN guidance for polymorbid medical inpatients suggests approximately 0.8 g/kg/day when eGFR is <30 mL/min/1.73 m^2^ without kidney replacement therapy [58]. This should not be applied as a universal ceiling in HF with frailty: targets must be reconciled with the distinction between stable CKD, acute illness and active catabolism outlined in Section 5, and the reference body weight must be specified [46,47]. Direct comparisons of higher versus lower protein provision in this population remain lacking [58].

An additional nutritional challenge concerns intentional weight loss in patients with obesity-related HFpEF and frailty. The STEP-HFpEF and SUMMIT trials demonstrated clinical benefits of incretin-based therapies in patients with obesity-related HFpEF [59,60,61,62]. A prespecified analysis of STEP-HFpEF also reported improvements in a deficit-based frailty index (OR 3.16; 95% CI 2.44–4.09), although these findings should not be interpreted as evidence of reversal of physical frailty or preservation of muscle mass [62]. From a nutritional perspective, the key challenge is to achieve beneficial adiposity reduction while preserving skeletal muscle and functional reserve. These findings should not be extrapolated to patients with malnutrition, cachexia, or HFrEF [59,62].

However, the most critical point of consensus between guidelines for heart failure (HF) and frailty remains the need to identify malnutrition and ensure adequate nutrient intake [3,53,55,58]. ESPEN recommends the early identification of malnutrition risk, the individualization of energy and protein targets, and the provision of adequate micronutrients, with targeted supplementation for confirmed or suspected deficiencies [58]. This approach must be distinguished from the indiscriminate supplementation of vitamins or trace elements, for which evidence in the context of HF remains limited [53]. Although current recommendations support individualized nutritional care, the effectiveness of specific interventions remains uncertain, particularly in patients with both HF and frailty. The available interventional evidence is critically examined in Section 7.

Consequently, modern management should shift away from a universal restrictive model toward phenotype-guided precision nutrition [3,53,54,55]. Patients with congestive HFrEF and cachexia or malnutrition, HF with frailty and CKD, HF with sarcopenia but without advanced renal dysfunction, or obesity-related HFpEF may require different priorities regarding sodium, fluids, energy, protein, and body weight [3,53,54,55,59]. These priorities should be reassessed as congestion, renal function, nutritional status, muscle function, and overall functional reserve change over time. Thus, the key unresolved question is no longer whether nutrition matters, but rather which phenotype should receive a specific intervention, at what intensity, and at what stage of the HF trajectory [7,55,63,64]. Future research should move beyond the assessment of individual nutrients and universal restrictions to test strategies that combine nutrition, exercise and rehabilitation, body composition monitoring, and contemporary HF treatment [55,63,65]. The ultimate goal is neither maximal restriction nor maximal intake, but rather maintaining HF control without accelerating malnutrition, muscle loss, and the loss of multisystem reserve.

## 7. Nutritional Interventions and Frailty-Related Outcomes

### 7.1. What Does the Current Evidence Actually Target?

Direct evidence on the effects of nutritional interventions on frailty in patients with heart failure remains limited and heterogeneous, both in terms of the type of intervention and the domains and endpoints assessed. In a systematic narrative review focused on nutritional interventions in older adults with HF and frailty-related outcomes, Belqaid et al. identified only eight studies, including seven controlled trials and one feasibility study. Interventions included vitamin D and protein supplementation, enteral nutrition or oral nutritional supplements, and a low-carbohydrate diet, whereas the outcomes primarily concerned body composition and functional measures related to frailty [7]. Broader evidence syntheses further highlight the uncertainty surrounding nutritional interventions in HF. A systematic review of 25 randomized trials found no significant mortality benefit from nutritional support alone, while evidence for combined nutrition and exercise was based on very small samples [63]. A more recent meta-analysis of 22 RCTs involving 4042 participants reported improvements in quality of life, walking distance, and grip strength, but no statistically significant reduction in all-cause mortality [66]. Substantial heterogeneity and methodological limitations preclude identification of an optimal intervention.

This heterogeneity is consistent with the broader landscape of interventions targeting frailty in HF. In the scoping review by Menoud et al., which included 155 reports and 44 unique interventions, only half of the interventions addressed at least two frailty domains. The physical-functional and clinical domains were targeted most frequently, with nutrition included within the clinical domain [67]. The available literature on nutrition and exercise further indicates that experimental studies assessing their combined effects on sarcopenia and frailty in HF remain scarce [65].

Consequently, available studies address different and partly overlapping levels of response to intervention, ranging from nutritional status and body composition to muscle mass and strength, physical performance, quality of life and, much less frequently, frailty status itself. These outcomes should not be regarded as equivalent, and improvement in a single domain does not in itself constitute evidence of reversal of frailty as an integrated syndrome. An additional interpretative limitation is the multicomponent nature of some interventions. In the randomized NICE-Support trial, improvements in frailty and quality of life were observed following a programme combining nutritional counselling, structured exercise, nursing consultations, and ongoing support. Because the intervention was evaluated as a whole, the independent contribution of its nutritional component cannot be isolated [68].

### 7.2. Muscle Mass, Strength, and Physical Performance

Muscle mass, strength, and physical performance represent related but non-interchangeable domains of the physical frailty phenotype, and available studies indicate that their responses to nutritional interventions in patients with HF may differ. A trial of 30 g/day whey protein isolate randomised 33 patients with chronic HF, of whom 25 completed 12 weeks. Improvements in skeletal muscle mass and its index were reported in the intervention group; however, the reported statistical analyses do not consistently establish a significant between-group treatment effect. Handgrip strength showed a borderline within-group change (*p* = 0.05), without convincing evidence of superiority over placebo [69]. A similar dissociation was observed in the study by Herrera-Martínez et al. After 24 weeks, lean body mass increased from baseline in the group receiving a high-energy, high-protein oral nutritional supplement, whereas handgrip strength did not change significantly and Timed Up and Go performance improved in both groups [70].

The absence of a close relationship between changes in body composition and function is also illustrated by other studies. In a double-blind RCT involving 31 patients with stable HF, supplementation with L-alanyl-L-glutamine and polyunsaturated fatty acids was associated with a modest increase in lean body mass in the active-treatment group, but did not improve handgrip strength, 6-min walk distance, or other measures of muscle function compared with placebo [71]. Similarly, in the study by Pineda-Juárez et al., adding 10 g/day of BCAA to resistance training and an individualized diet did not provide additional improvements in muscle strength or exercise capacity. Improvements in these outcomes were observed in both groups, suggesting a greater contribution from the training programme than from BCAA supplementation itself [72].

Functional outcomes were not uniformly neutral, however. In a small randomized study involving 38 older patients with stable HF and substantial muscle depletion, supplementation with 8 g/day of essential amino acids was associated with within-group improvements in exercise power, peak VO_2_, and 6-min walk distance, which were not observed in controls. However, the small sample and limited reporting of between-group treatment effects warrant cautious interpretation. Changes in arm muscle area were similar between groups [73].

In a pilot HFpEF trial, 23 patients were randomised, 17 completed follow-up and 16 were analysed after exclusion of an outlier. Protein plus exercise improved walking performance, whereas protein alone did not; quadriceps strength also increased with protein alone [74]. The small sample, one-sided testing and absence of an exercise-only arm preclude firm conclusions about an additional protein effect beyond exercise.

Taken together, these findings indicate that nutritional interventions may modify selected muscle-related and functional domains associated with frailty in HF; however, increases in muscle quantity do not consistently translate into greater strength or better physical performance. Functional response may also depend on the patient’s baseline phenotype, the nutritional intervention used, and concomitant physical activity. Because most of these studies did not recruit patients on the basis of formally diagnosed frailty and did not assess frailty as an integrated endpoint, improvements in individual domains should not be interpreted as evidence of frailty reversal.

### 7.3. Individualized Nutritional Support and Frailty-Related Outcomes

Evidence from the EFFORT trial provides support for individualized nutritional management in hospitalized patients with HF at nutritional risk. In a subgroup analysis involving 645 patients, protocol-driven nutritional support targeting energy, protein, and micronutrient requirements was associated with improved outcomes compared with standard hospital nutrition [51]. However, this subgroup analysis did not specifically evaluate patients with formally diagnosed frailty and should not be interpreted as evidence supporting a particular nutritional prescription for this population.

Individualised nutritional support may improve nutritional status, but functional and clinical effects remain uncertain. In the 86-patient PACMAN-HF trial, nutritional intervention reduced the composite risk of death or HF hospitalisation (HR 0.39; 95% CI 0.19–0.83) [75]. A prespecified 64-patient subanalysis reported improved nutritional status, but its clinical estimate was imprecise (HR 0.34; 95% CI 0.11–1.09) [76]. Walking distance increased within the intervention group; this alone does not establish a between-group effect. The parent trial and subanalysis represent overlapping evidence.

Improvement in nutritional status does not necessarily lead to superior physical performance. In the randomized trial by Guerra-Sánchez et al., individualized nutritional counselling combined with nutritional supplementation improved nutritional status in patients with chronic HF, whereas no similarly clear advantage of the intervention was demonstrated for functional outcomes [77]. This distinction is important because correction of nutritional vulnerability and improvement in physical performance represent related but separate therapeutic goals.

The more recent multicentre BOCADOS-IC trial provides further evidence regarding domains related to the frailty phenotype. Among 204 patients aged >65 years with chronic HF and an MNA-SF score ≤ 11, a structured strategy comprising individualized dietary optimization and physical activity recommendations resulted in greater improvements in MNA-SF score, mid-arm muscle circumference, and HF-related quality of life than usual care. No significant difference in death or HF hospitalisation was detected at 6 months (HR 0.90; 95% CI 0.48–1.70); recruitment stopped early, limiting conclusions about clinical efficacy [78]. Because the intervention also included physical activity recommendations, changes in muscle-related outcomes cannot be attributed exclusively to nutritional management [78].

Additional evidence comes from a smaller randomized study involving 90 older patients with chronic HF, in which an individualized dietitian-led nutritional prescription based on a food-exchange approach was associated with improvements in measures of cardiorespiratory performance, including the 6MWT, whereas several parameters related to muscle mass did not differ significantly between groups [79].

Overall, these findings indicate that improvement in nutritional status may, but does not necessarily, lead to better physical performance or a more favourable muscle phenotype. Importantly, none of these trials assessed frailty as an integrated primary endpoint. Their findings should therefore be interpreted as evidence relating to selected frailty-related domains rather than as evidence that individualized nutritional support reverses frailty as a clinical syndrome.

### 7.4. Nutrition as Part of Multimodal Frailty Care

The NICE-Support trial randomised 240 patients with stable HF to a 12-week programme combining nutritional counselling, exercise, nursing consultations and ongoing support, or to usual care; 192 completed the 24-week assessment. Frailty and quality of life improved more with the programme (Cohen’s d 1.17 and 0.87, respectively) [68]. Because the components were delivered together, the independent nutritional contribution cannot be determined.

REHAB-HF randomised 349 older patients hospitalised for acute decompensated HF to multidomain rehabilitation (*n* = 175) or usual care (*n* = 174). The intervention improved the SPPB score by 1.5 points at 3 months, without a significant reduction in rehospitalisations at 6 months [80]. In the prespecified frailty analysis (*n* = 337), the SPPB treatment effect was larger in frail than prefrail participants (2.1 versus 0.8 points; *p* for interaction = 0.03) [81]. These findings support rehabilitation within multimodal care but do not establish a nutritional treatment effect.

Taken together, the available evidence encompasses nutrition-only interventions, combined nutrition and exercise strategies, and broader multidomain programmes. Although multimodal approaches appear promising for improving frailty-related outcomes, their superiority over isolated nutritional interventions has not been conclusively established. Moreover, the independent contribution of nutritional therapy cannot be determined from multicomponent trials. These findings support considering nutritional care within phenotype-directed, multidisciplinary strategies while acknowledging the limited and heterogeneous evidence [7,65,82].

### 7.5. Clinical Interpretation and Key Take-Home Messages

Interpretation of the effectiveness of nutritional interventions in patients with HF and frailty requires distinguishing the level at which treatment response is observed. Improvement in nutritional status, body composition, muscle mass, muscle strength, physical performance, and frailty status itself represents related but distinct clinical outcomes [3,83,84]. Accordingly, an increase in body weight or lean body mass should not be interpreted as evidence of improved muscle function, just as an improvement in handgrip strength or 6-min walk distance does not in itself demonstrate a reduction in frailty severity. Frailty, sarcopenia, cachexia, and malnutrition frequently coexist in patients with HF, but they remain distinct clinical constructs and require separate assessment [3,83,84].

The available evidence therefore supports interpreting the effects of nutritional interventions in relation to the individual patient phenotype. The clinically relevant question is not merely whether nutritional support “works” in patients with HF and frailty, but which potentially modifiable domain is being targeted, in which patient phenotype, and whether improvement at that level translates into meaningful functional or clinical benefit. Importantly, frailty should not be regarded solely as a marker of poor prognosis or as a reason to withhold intervention. Available HF data indicate that patients with frailty may retain substantial capacity for functional improvement, although direct evidence from randomized trials assessing frailty itself as an endpoint remains limited [4,62].

Future trials should therefore incorporate standardized frailty assessment alongside evaluation of nutritional status, body composition, physical function, and patient-centred outcomes. This would help determine whether improvement in individual domains truly translates into modification of the broader frailty phenotype rather than merely into changes in selected surrogate measures [84,85].

In patients with HF and frailty, nutritional assessment should account for congestion, weight trajectory, muscle status, and functional limitations rather than relying on a single screening measure. Energy and protein targets should be individualized and reassessed as clinical conditions change, particularly in the presence of renal dysfunction or nutritional depletion. Nutritional interventions should be considered alongside physical activity or rehabilitation when feasible, while recognizing that improvements in nutritional or muscular parameters do not necessarily indicate reversal of frailty. Given the limited direct evidence, these principles should guide individualized clinical judgment rather than be interpreted as validated treatment algorithms.

## 8. Evidence Gaps and Research Priorities

Despite growing interest in nutritional care in heart failure (HF), evidence specifically addressing patients with concomitant frailty remains limited and fragmented. Future studies should move beyond heterogeneous populations and isolated nutritional endpoints towards phenotype-specific strategies that distinguish frailty, malnutrition, sarcopenia, cachexia, and obesity-related HF. Standardized definitions and harmonized assessment frameworks are needed to improve comparability across studies.

A major priority is to establish clinically relevant energy and protein targets according to HF phenotype, frailty severity, renal function, congestion status, and body composition. Trials should also determine whether nutritional interventions are most effective when delivered alone or as part of multimodal programmes incorporating resistance exercise, rehabilitation, and multidisciplinary support.

Importantly, future research should avoid relying solely on body weight or lean mass as surrogate outcomes. Core outcome sets should integrate nutritional status, muscle strength, physical performance, validated frailty measures, quality of life, hospitalization, and mortality. Longer follow-up is also required to determine whether short-term improvements translate into sustained functional or clinical benefit.

Another important research priority concerns emerging therapies that induce substantial weight loss in obesity-related HF, particularly how to reduce adiposity while preserving skeletal muscle, physical function, and physiological reserve.

Implementation represents an additional research priority. A recent scientific statement emphasizes the importance of individualized nutritional assessment and multidisciplinary care involving dietitians and other healthcare professionals [86]. However, barriers related to access to nutritional services, multidisciplinary coordination, and continuity of care may hinder the implementation of these strategy [5]. Future research should therefore evaluate not only the effectiveness of nutritional interventions but also their feasibility, accessibility, and implementation within routine HF care.

## 9. Conclusions

Nutritional care in patients with HF and frailty requires an individualized, phenotype-based approach rather than a uniform dietary strategy. Frailty, malnutrition, sarcopenia, and cachexia frequently coexist but represent distinct clinical constructs and should be assessed separately. No single screening tool, anthropometric measure, or prognostic index adequately captures nutritional vulnerability in this population, particularly in the presence of congestion and altered body composition. Current evidence supports early identification of nutritional risk, individualized energy and protein provision, and integration of nutritional care with physical activity or rehabilitation when feasible. However, these recommendations are based on a combination of direct and extrapolated evidence, and no universally validated nutritional prescription exists for patients with both HF and frailty. Optimal targets for energy, protein, sodium, fluids, and weight management therefore remain uncertain and depend on HF phenotype, renal function, nutritional status, and treatment goals. In obesity-related HFpEF, intentional weight loss may improve symptoms and function, but preservation of skeletal muscle and physical reserve remains essential. Overall, nutritional management should aim not only to correct nutritional deficits, but also to preserve function, muscle integrity, and physiological reserve. Multidisciplinary, phenotype-directed care appears most consistent with the complexity of patients with HF and frailty. Future trials should evaluate phenotype-specific nutritional strategies using standardized frailty definitions and clinically meaningful outcomes, including physical function, quality of life, hospitalization, and mortality.

## Figures and Tables

**Table 1:** Nutritional screening and assessment approaches relevant to patients with heart failure and frailty *.

Tool/Approach	Type	Main Components	Evidence/Use in HF	Potential Relevance to Frail HF	Key Limitations in Frail HF
NRS-2002 [21]	Nutritional screening	BMI, recent weight loss, reduced food intake, disease severity; age adjustment in older adults	Frequently used in hospitalized populations, including patients with HF	Rapid and widely available; incorporates disease severity and reduced intake	Body weight and BMI may be distorted by congestion; limited specificity for frailty-related nutritional impairment
MUST [5,16]	Nutritional screening	BMI, unintentional weight loss, acute disease effect	Used across hospital and community settings; HF-specific validation is relatively limited	Simple, rapid and inexpensive; suitable for routine screening	Strong dependence on body weight and BMI; fluid retention may mask weight loss and malnutrition
MNA-SF [22]	Nutritional screening	Reduced food intake, weight loss, mobility, psychological stress or acute disease, neuropsychological problems, BMI or calf circumference	Extensively used in older adults and increasingly evaluated in older patients with HF	Captures mobility and other domains closely related to geriatric vulnerability and frailty	Partial conceptual overlap with frailty constructs; BMI and calf circumference may be affected by oedema or altered body composition
MNA [22,25]	Comprehensive nutritional assessment	Anthropometric, general, dietary and subjective domains	Evaluated in older HF populations and associated with adverse clinical outcomes	Particularly relevant in older and functionally impaired patients; captures broader nutritional and functional context	More time-consuming than brief screening tools; some domains overlap with frailty and functional status
SGA [25]	Comprehensive nutritional assessment	Weight and dietary history, gastrointestinal symptoms, functional capacity, disease burden, physical signs of muscle and fat loss, oedema/ascites	Applied in HF and other chronic disease populations	Allows integration of clinical history with examination of muscle and fat stores	Examiner dependent; subjective elements reduce reproducibility; oedema may complicate interpretation
GNRI [17,22,28]	Nutritional risk/prognostic index	Serum albumin and ratio of current to ideal body weight	Extensively studied in acute and chronic HF; consistently associated with prognosis in many cohorts	Simple, objective and based on routinely available clinical data	Albumin is influenced by inflammation, congestion and hepatic dysfunction; body weight is affected by fluid status; better regarded as a risk index than a diagnostic tool for malnutrition
CONUT [17,22,23,28]	Prognostic nutritional index	Serum albumin, total cholesterol and total lymphocyte count	Widely investigated as a prognostic marker in HF	Objective, inexpensive and based on routinely available laboratory measurements	Influenced by inflammation, disease severity, immune status and lipid-lowering therapy; may poorly reflect muscle-related nutritional impairment.
PNI [22,28]	Prognostic nutritional index	Serum albumin and total lymphocyte count	Frequently evaluated in HF and associated with mortality and other adverse outcomes	Simple and readily calculable from routine laboratory data	Reflects inflammatory and disease-related processes as well as nutritional status; lacks direct assessment of weight loss, dietary intake and muscle mass
GLIM criteria [24,26,27]	Diagnostic framework for malnutrition	At least one phenotypic criterion: weight loss, low BMI or reduced muscle mass; plus at least one etiologic criterion: reduced intake/assimilation or inflammation/disease burden	Increasingly studied in HF populations, including older and hospitalized patients	Particularly relevant because reduced muscle mass and disease burden can be incorporated into diagnosis	Requires prior nutritional screening; assessment of muscle mass may require additional techniques; congestion may obscure weight-based criteria; optimal implementation in frail HF remains uncertain

* Selected references illustrate the use and performance of individual approaches across hospitalized, outpatient, and older HF populations. These tools serve different purposes, including nutritional screening, comprehensive assessment, diagnosis, and prognostic evaluation, and should not be considered interchangeable diagnostic measures of malnutrition. Their applicability depends on the clinical setting and patient characteristics.

## Data Availability

The original contributions presented in this study are included in the article. Further inquiries can be directed to the corresponding author.

## Abbreviations

The following abbreviations are used in this manuscript:

ACC	American College of Cardiology
BCAA	Branched-Chain Amino Acids
BIA	Bioelectrical Impedance Analysis
BMI	Body Mass Index
CKD	Chronic Kidney Disease
CONUT	Controlling Nutritional Status
DXA	Dual-Energy X-ray Absorptiometry
ESPEN	European Society for Clinical Nutrition and Metabolism
GDF-15	Growth Differentiation Factor 15
GLIM	Global Leadership Initiative on Malnutrition
GLIS	Global Leadership Initiative in Sarcopenia
GNRI	Geriatric Nutritional Risk Index
HF	Heart Failure
HFpEF	Heart Failure with Preserved Ejection Fraction
HFrEF	Heart Failure with Reduced Ejection Fraction
JCS	Japanese Circulation Society
JHFS	Japanese Heart Failure Society
KCCQ-CSS	Kansas City Cardiomyopathy Questionnaire Clinical Summary Score
KRT	Kidney Replacement Therapy
MNA	Mini Nutritional Assessment
MNA-SF	Mini Nutritional Assessment–Short Form
MUST	Malnutrition Universal Screening Tool
NRS-2002	Nutritional Risk Screening 2002
NYHA	New York Heart Association
PNI	Prognostic Nutritional Index
REE	Resting Energy Expenditure
SGA	Subjective Global Assessment
SPPB	Short Physical Performance Battery
TEE	Total Energy Expenditure
6MWT	6-Minute Walk Test
